# Multiple abiotic stress, nitrate availability and the growth of wheat

**DOI:** 10.1016/j.still.2019.04.005

**Published:** 2019-08

**Authors:** Y. Ge, M.J. Hawkesford, C.A. Rosolem, S.J. Mooney, R.W. Ashton, J. Evans, W.R. Whalley

**Affiliations:** a Rothamsted Research, Harpenden, AL5 2JQ, United Kingdom; b São Paulo State University, Botucatu, Brazil; c School of Biosciences, University of Nottingham, Leicestershire, PC, United Kingdom

**Keywords:** Leaf stunting, Root impedance, Nutrient stress

## Abstract

In the field, wheat experiences a combination of physical and nutrient stresses. There has been a tendency to study root impedance and water stress in separation and less is known about how they might interact. In this study, we investigated the effect of root impedance on the growth of three wheat varieties (Cadenza, Xi19 and Battalion) at different levels of nitrate availability, from 0–20 mM nitrate, in sand culture. This model system allows soil strength to be increased while maintaining adequate water availability. In a separate pot experiment, we grew the same wheat varieties in a loamy sand where soil was allowed to dry sufficiently to both reduce water potential and increase root impedance. This pot experiment also had a range of nitrate availabilities 0–20 mM nitrate. Once the seedlings were established we limited water supply to apply a matric potential of approximately −200 kPa to the roots. Soil drying increased the penetrometer resistance from approximately 300 kPa to more than 1 MPa. There were differences between the two experimental systems; growth was smaller in the soil-based experiment compared to the sand culture. However, the effects of the experimental treatment, root impedance or water withholding, relative to the control were comparable. Our data confirmed that leaf elongation in Cadenza (carrying the tall *Rht* allele) was the most sensitive to root impedance. Leaf stunting occurred irrespective of nitrate availability. Leaf elongation in the Xi19 and Battalion (carrying the semi-dwarf *Rht* allele) was less sensitive to root impedance and drought than Candenza. We suggest that the critical stress in a pot experiment where the soil was allowed to dry to approximately −200 kPa was root impedance and not water availability.

## Highlights

• Leaf elongation is lower when roots are impeded irrespective of nitrate supply.
• Root impedance and soil drying have a similar impact on root and shoot grow.
• We found genotypic effects in leaf stunting due to abiotic stress.
• Root diameter decreases with decreasing nitrate availability.
• Root diameter increases with root impedance and moderate soil drying.

## 1 Introduction

While the response of crops to nutrients, in particular nitrogen, has been studied in an agronomic context (e.g. Lawlor et al., 1981), less is known about how nutrient and abiotic stresses interact to determine yield. Soil compaction is associated with both decreased nitrogen uptake and yield (Alakukku and Elonen, 1995). Poor root growth in compacted soils is commonly used as at least a partial explanation. It is widely assumed that the effects of compact soil are simply to limit nitrogen availability either by decreased capture by roots or denitrification in water logged soils (e.g. Chamen et al., 2015). However, stunting of crop growth in compacted soil by hormonal signalling is also likely to contribute to lower yields and this can occur even when nutrient and water supply are adequate (Masle and Passioura, 1987). This is thought to be related to the effects of root impedance. However, it is not clear if nutrient availability affects stunting of crops due to root impedance. The roots of wheat, or indeed any plant, growing a field will extract water and dry the soil (Passioura, 1991). As the soil dries water becomes less available as matric potential decreases and soil strength (or root impedance) increases (Gao et al., 2016). Mirreh and Ketcheson (1973) concluded that for any given level of penetration resistance the elongation of maize roots was further reduced by decreasing matric potential, however, this study was concerned with seedlings no older than 36 h and hence with a minimal water uptake. When soybean was grown for 5 days, Yapa et al. (1988) concluded that although penetration resistance represents an integration of bulk density and water content, an improved prediction of root penetration into soil cores was possible if the separate components (penetration resistance, density and water content) were considered. To investigate the effects of root impedance in isolation from water stress, sand culture systems (Collis-George and Yoganathan, 1985; Coelho Filho et al., 2013; Jin et al., 2015a; Whalley et al., 2013) have been used, which isolate the effects of high soil strength from water stress. Mechanical impedance can be increased by increasing the confining pressure on a column of sand without any effect on the water or nutrient availability. These experiments replicate the effect of root impedance by soil compaction in the field (Atwell, 1990) and in pot experiments (Masle and Passioura, 1987) on the tiller number and leaf elongation. Increased root impedance lowers the number of tillers and the elongation of leaves. Even in relatively moist soil the impedance to root elongation can be high (Whalley et al., 2006). After only a limited amount of soil drying there is an increase in root impedance (Whalley et al., 2007), thus the sand culture systems replicate the physical environment in a field during early growth, when there has been limited water uptake, or when the soil is well-watered. However, pot experiments have been never compared with sand culture experiments.

In a sand culture system, a column of sand is placed in a tank containing nutrient solution a capillary fringe will be established approximately 10 cm above the water table and the saturation of the sand will depend on the height above the water table. Wheat roots tend to occupy the unsaturated sand between the capillary fringe and the top of the sand column (e.g. Jin et al., 2015a) and they are considerably shorter roots in comparison with to those from soil-filled rhizotrons (Jin et al., 2015b). While the advantage of the sand culture is a precise control of the physical aspects of the root environment, and the data can be simply interpreted in terms of a single abiotic stress (i.e. root impedance), it is open to criticism because it may not reflect what roots experience in drying soil. Pot experiments, using field soil, provide more complex rooting environment and depending on the degree of soil drying that is permitted, they can be used to explore how plants respond to combinations of water stress and root impedance (Whalley et al., 2000). The disadvantages of pot experiments are summarised by Passioura (2006), who notes that a significant problem is that potted soil, when wet, can be prone to hypoxia. When the soil dries, roots are exposed to multiple stresses (i.e. water availability and root impedance) and it is difficult be certain about the identity of the key growth limiting stress, or what combination of stresses impact most strongly on plant growth.

In this paper, we describe a comparison of the growth of three wheat varieties in a sand culture system and in a pot experiment where water was withheld. It is necessary to use two different growth systems to obtain different combinations of abiotic stresses associated with root impedance and water availability. In the sand culture system root impedance can be increased while maintaining adequate water supply, aeration and nutrient supply. This allows the effects of root impedance on plant growth to be studied in isolation. In pot experiments the effects of soil drying can be studied where both water stress and root impedance both increase with soil drying. It is possible that by comparing both approaches we can obtain a greater insight into the response of wheat to abiotic stress. Apart from soil strength and water availability, root architecture responds to nutrient status and in particular nitrogen (Forde, 2014). Therefore, with both experimental systems we investigated the interaction between nitrate availability and abiotic stress.

## 2 Materials and methods

### 2.1 Plant material

Three wheat varieties (Cadenza, Xi19 and Battalion) were used in this study. Our previous work (Jin et al., 2015a; Coelho Filho et al., 2013) found that leaf elongation in Cadenza, containing a tall *Rh*t allele, is more sensitive to root impedance in comparison with semi-dwarf wheats. Xi19 and Battalion are semi-dwarf varieties which contain the semi dwarf *Rht* alleles.

### 2.2 Experiment 1: Root impedance at different nitrate concentrations

**Fig. 1:**
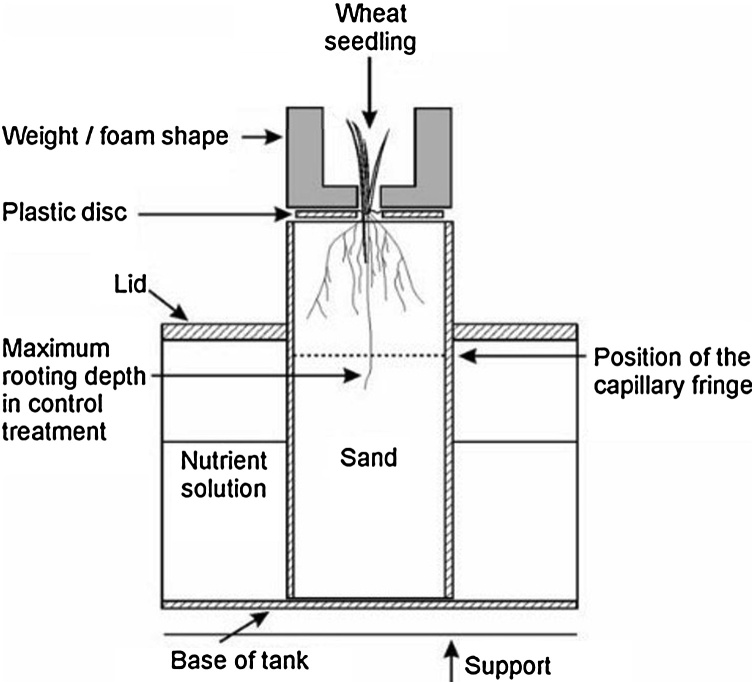
Schematic representation of the experimental sand culture growth system.

The sand culture system (Fig. 1) was used to investigate the effects of strong soil in isolation of other abiotic stresses as described previously (Jin et al., 2015a; Whalley et al., 2006; Coelho Filho et al., 2013). Each sand-core apparatus consisted of an aluminium tank containing six sand-filled tubes in a 3 × 2 arrangement. We used rigid plastic tubes 45 cm long and 15 cm in diameter to contain the sand. The tubes were supported 45 mm above the base of the tanks on aluminium mesh, covered with nylon cloth. Dry sand (RH65 grade; Double Arches Quarry/Eastern Way, Leighton Buzzard LU7 9LF, UK) was poured into the tubes together with nutrient solution. We used a template to give a level surface raised 8 mm above the top of the tube. The sand columns were allowed to drain to equilibrium overnight and then covered with plastic discs 3 mm thick and 14 cm in diameter. The water table height was maintained at 30 cm below the surface of the sand. Two levels of impedance were tested: impeded and control. A steel mass of 17 kg was placed on the plastic disc to achieve the high impedance, while the control used a mock weight made of foam to simulate the physical environment around the shoot due to the steel weight. The steel weight and foam produced the penetrometer resistance of approximately 0.75 and 0.19 MPa, respectively (Whalley et al., 1999). This penetrometer resistance of 0.75 MPa is not particularly high and typically found in soil during the early stages of soil drying (Whalley et al., 2007), however, it is sufficiently high to affect the growth of wheat (Jin et al., 2015a). The compressibility of the sand we used was minimal, under these loads, and the application of the load had a minimal effect on density. It did however, increased the confining pressure and make it harder to expand cavities.

The highest nitrate concentration was 20 mM nitrate (N20) and it contained 10 mM Ca(NO_3_)_2_, 1 mM KH_2_PO_4_, 4.0 mM KCl, 2.0 mM MgSO_4_, 4.0 mM CaCl_2_.2H_2_O, with the following micronutrients: 60 μM Si, 50 μM B, 50 μM Fe, 15 μM Mn, 0.8 μM Zn, 0.3 μM Cu and 0.1 μM Mo. In the other nitrogen treatments, the concentrations of Ca(NO_3_)_2_ were 5 mM Ca(NO_3_)_2_; 2.5 mM Ca(NO_3_)_2_; 1 mM Ca(NO_3_)_2_; 0.5 mM Ca(NO_3_)_2_ to give N10, N5, N2, N1 which corresponded to 10, 5, 2 and 1 mM nitrate. In addition, we included a treatment without any nitrate. The nutrient solution in the tanks was replaced 21 days after the start of the experiment.

Seeds were germinated between two sheets of wet filter paper in Petri dishes which were wrapped in aluminium foil to exclude light. Wheat seedlings were transplanted into the top of the sand column into a 2 cm deep hole (created with a pencil) in the centre of each tube. All of the seminal roots were shorter than 1 cm. The experiments were conducted in a controlled environment room. The day and night temperatures were 22 and 18 °C, respectively, with a day length of 14 h. The relative humidity was 70% during the day and 80% at night. Lighting was by fluorescent tubes, with supplementary tungsten lighting, and photosynthetic photon flux density was 450 μmol m^−2^ s^−1^ at plant height.

Due to a limited number of sand columns, the complete experiment was repeated three times. In each experiment the experimental treatments were 3 wheat varieties × 2 levels of root impedance × 6 nitrate levels, to give 36 treatment combinations.

When roots are smaller than the pore-sizes in sand culture, then this model system offers no impedance to root elongation, despite the application of impedance with the steel mass. For example, Whalley et al. (1999) found that fine carrot roots were not impeded by sand culture, but thicker onion roots were. Wheat roots have a diameter of approximately 0.5 mm, and these roots will be impeded by sand culture. In previous studies (Jin et al., 2015a; Coelho Filho et al., 2013; Whalley et al., 2006) this approach has been effective at impeding wheat roots.

### 2.3 Experiment 2: Soil drying at different nitrate concentrations

In a pot experiment, we compared the growth of the three wheat varieties in drying soil with growth in well-watered conditions. We used soil from Butt Close experimental field, Woburn Experimental Farm (52°00′42″N, 0°32′42″W), Rothamsted Research, UK. Butt Close soil is a loamy sand soil (sand: 87.5%, silt: 5.5% and clay: 7.2%), and taxonomically these soils are classified as Cambic Arenosols(FAO series). This soil is low in organic carbon (1%), near neutral in soil pH (6.63, 1:2 soil water ratio), with a particle density of 2.65 g cm^−3^ (Whalley et al., 2008). The nitrogen concentration of the soil was 0.095 ± 0.0045%, measured by dry combustion with a LECO TruMac combustion analyser (LECO Corp., St Joseph, MI). This is a very low N concentration, and comparable with a silty clay loam soil which had been left in a fallow condition for 49 years at Rothamsted Research (Gregory et al., 2016).

We used cubic pots approximately 8 cm tall and 460 cm^3^ in volume containing 500 g of dry loosely packed (approximately 1.35 g cm^−3^) Butt Close soil. The water content of the soil was adjusted to approximately 24 g cm^−3^, using an appropriate nutrient solution. This gave an air-filled porosity of approximately 15% and a matric potential of −10 kPa. Three seeds were sown in each pot. When the first leaf emerged, seedlings were thinned to one per pot and watered daily at about 15:00 h to maintain the initial, well-watered condition until the emergence of leaf five (after approximately two weeks). Thereafter, we continued to water the well-watered control treatment, but stopped watering the drought treatment until the water content was approximately 7.5 g cm^−3^ to give a matric potential of approximately −200 kPa. All of the pots were covered with a 2 cm layer of black plastic beads to minimize evaporation from soil surface. We applied the same N treatments, as used in the sand culture, with three replicates of each treatment combination (i.e. 3 replicates × 2 water regimes × 6 N treatments). Pots were weighed daily and adjusted to the required water content, described above, by slowly adding the nutrient solution onto the soil surface. This experiment was also conducted in a controlled environment using exactly the same environmental conditions as the first experiment.

### 2.4 Plant measurements

We took daily measurements of the length and width of the first 7–8 leaves on the first tiller, using a Perspex ruler. The SPAD (a registered trade mark owned by Minolta) meter value of the leaves was measured with a hand-held meter daily and provides a simple, quick, and non-destructive method for estimating leaf chlorophyll content. SPAD readings can be used to give an indication of the nitrogen content of the leaves (Lopez-Bellido et al., 2004) and as such provide a useful non-destructive way to monitor leaf nitrogen during the experiment. After harvest the nitrogen content of the shoots was measured with a Leco combustion analyser.

At harvest (40 days for experiment 1 and 30 days for experiment 2) the number of tillers and nodal root axes were counted, and the maximum depth of root was measured. Roots were washed free of sand or soil and spread out in water with minimal overlap. Root diameter and length were estimated using WinRHIZO (Regent Instruments, Quebec, Canada) in grey scale at 400 dots per inch (dpi) with a filter of 1.0 mm^2^. Root diameters (d) were recorded in 31 classes between 0 and 3.0 mm, which were bulked into 10 groups: 0 < d ≤ 0.1, 0.1 < d ≤ 0.2, 0.2 < d ≤ 0.3, 0.3 < d ≤ 0.4, 0.4 < d ≤ 0.5, 0.5 < d ≤ 0.6, 0.6 < d ≤ 1.0, 1.0 < d ≤ 1.5, 1.5 < d ≤ 2.0 and d>2.0 mm. After roots were scanned, they were oven dried at 70 °C for 48 h to measure dry weight.

### 2.5 Soil penetration resistance

Just before the soil-grown plants were harvested, a cone penetrometer with 30° cone angle and a basal diameter of 2 mm was used to make penetrometer resistance measurements to a depth of 50 mm below the soil surface. After the penetrometer resistance was measured and the soil water content was determined by oven drying a subsample of soil.

### 2.6 Statistical analysis

To analyse our data, we used Genstat V19 (VSN International Ltd. 5 The Waterhouse, Waterhouse street, Hemel Hempstead, HP1 1ES, UK) which gave a standard error of differences (SED) to allow comparison between any two means as well as the standard error of the mean (SE) (Webster, 2007). In the sand culture experiment the treatment factors were 3 wheat varieties, 2 levels of root impedance and 6 nitrate levels which gave 36 treatment combinations and a treatment structure of “wheat variety × root impedance × nitrate level”. The block structure was replicate/tank/pot; the complete experiment was repeated three times.

In the soil drying experiment the treatment factors were 3 wheat varieties, 2 levels of soil water and 6 nitrate levels. There were three replications in a single experiment to give 108 pots. We used the treatment structure “wheat variety × water availability × nitrate level” and the block structure “block/pot” in the ANOVA of these data.

Statistical analysis of the leaf elongation measurements was done by modelling the general response as a linear regression and then superimposing the approximate sigmoid shape over time using varieties all in the context of REML. Separate splines were used for each leaf and for each treatment combination. This approach was adopted as the exact form of non-linear response over time was not important. This approach was used to analyse similar leaf elongation data by Jin et al. (2015a).

## 3 Results

### 3.1 Leaf elongation

**Fig. 2:**
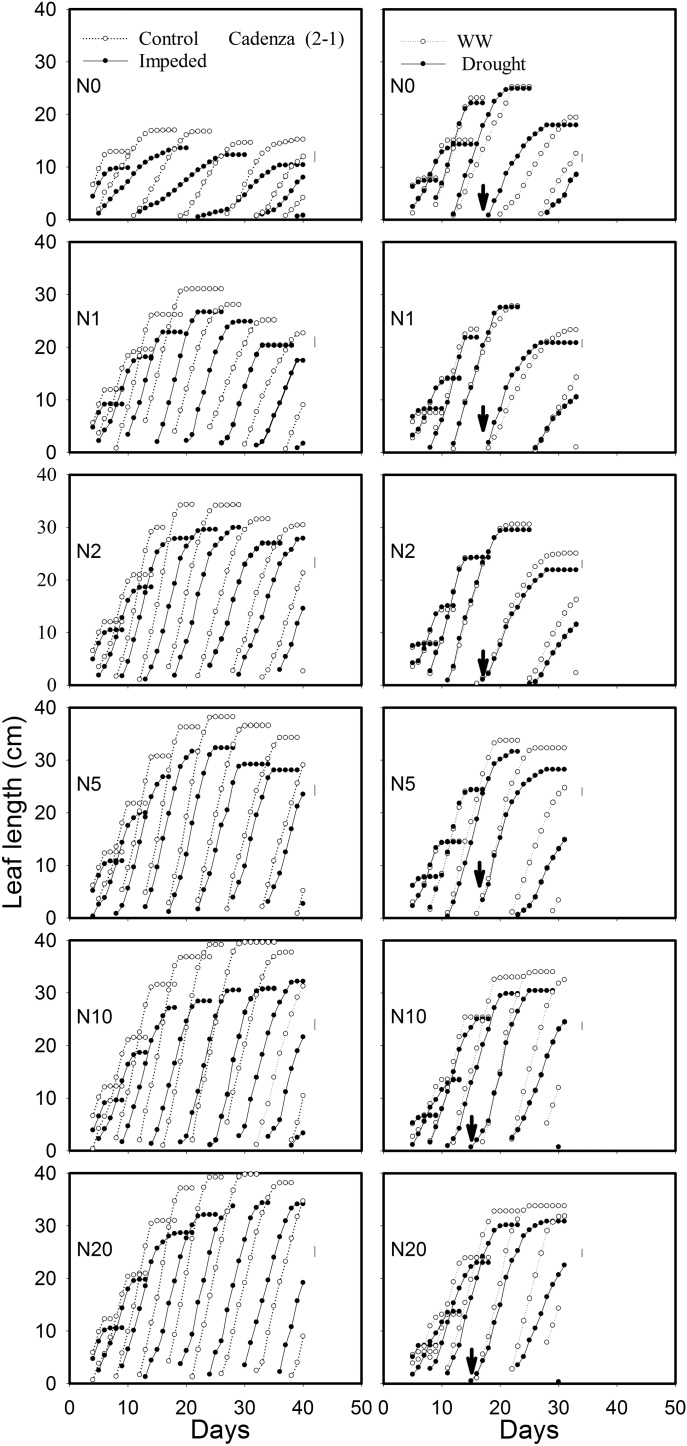

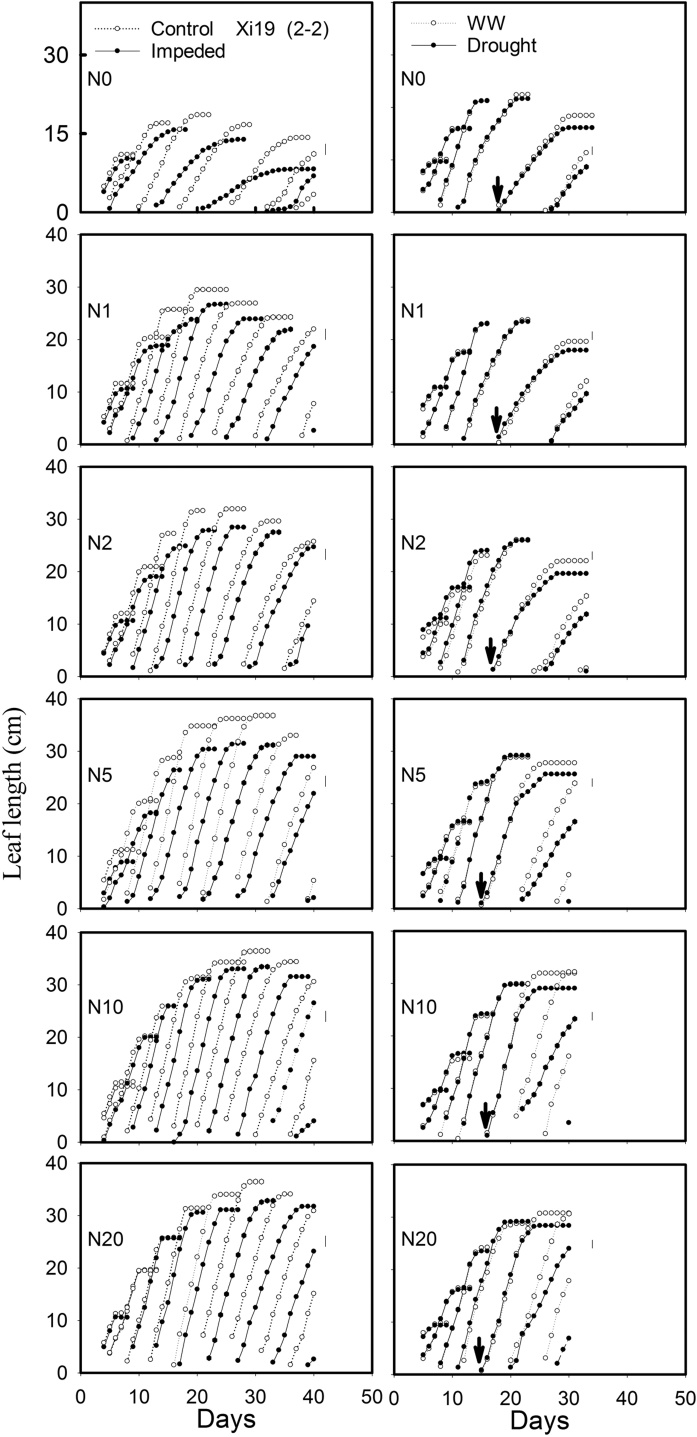

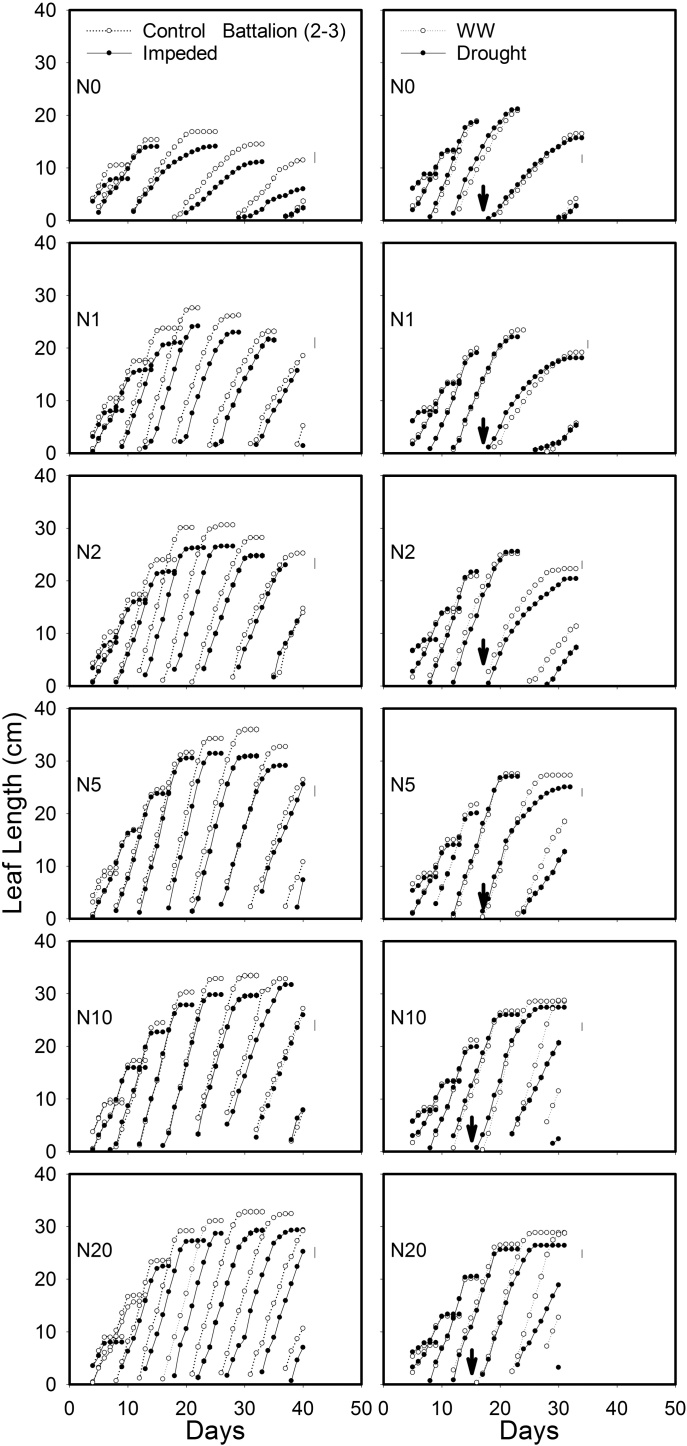
(1) The effects of nitrogen supply on leaf elongation (leaves1 up to 9, depending on treatment) in the low impedance control and mechanically impeded treatments (Left) and in the well-watered and drought treatments(right) for Cadenza. The arrow represents the date where irrigation was limited in the drought treatment. Data are means of three replications. The main effects of root impedance, water treatments and nitrogen supply significant effect at P < 0.001. In each graph, leaf number 1 is on the right and data for successive leaves is plotted sequentially. For each leaf number, two sets of data appear: the control and treatment as indicated in the top panels. (2) The effects of nitrogen supply on leaf elongation (leaves1 up to 9, depending on treatment) in the low impedance control and mechanically impeded treatments (Left) and in the well-watered and drought treatments(right) for Xi19. The arrow represents the date where irrigation was limited in the drought treatment. Data are means of three replications. The main effects of root impedance, water treatments and nitrogen supply significant effect at P < 0.001. In each graph, leaf number 1 is on the right and data for successive leaves is plotted sequentially. For each leaf number, two sets of data appear: the control and treatment as indicated in the top panels. (3) The effects of nitrogen supply on leaf elongation (leaves1 up to 9, depending on treatment) in the low impedance control and mechanically impeded treatments (Left) and in the well water and drought treatments (right) for Battalion. The arrow represents the date where irrigation was limited in the drought treatment. Data are means of three replications. The main effects of root impedance, water treatments and nitrogen supply significant effect at P < 0.001. In each graph, leaf number 1 is on the right and data for successive leaves is plotted sequentially. For each leaf number, two sets of data appear: the control and treatment as indicated in the top panels.

**Fig. 3:**
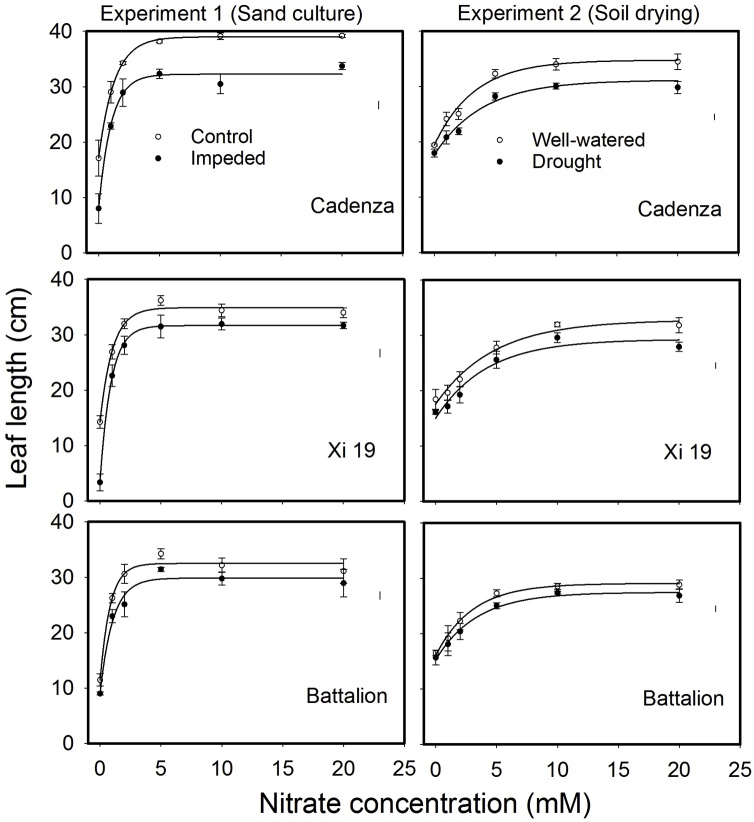
The final length of leaf 5 in three cultivars in the root impedance and the control treatments of the sand culture experiment and in the well-watered and drought treatments of the pot experiment.

Leaf elongation data and the final length of leaf five, for both the experiments, are shown in Figs. 2 (1)–(3) and 3 . The lengths of leaves 1–5 were stunted by root impedance, and leaf stunting appeared to be the greatest in Cadenza. The leaf length in Xi19 and Battalion seemed to be less sensitive to root impedance when nitrate levels were greater than 10 mM. In the sand culture experiment there was a significant three-way interaction of root impedance × wheat varieties × nitrate on final leaf length for leaf 4, leaf 5 and leaf 6. (*P* = 0.02, F_10,60_ = 2.35 for leaf 4, *P* = 0.006, F_10,60_ = 2.83 for leaf5 and *P* = 0.011, F_10,60_ = 2.73 for leaf6). In the soil drying experiment, once the drought treatment had been applied, we observed leaf stunting in all three wheat varieties (*P* < 0.001, F_1,48_ = 87.86 for leaf 5). Again, Cadenza appeared to be more sensitive to leaf stunting than either Xi19 or Battalion (Figs. 2 (1)–(3) and 3). There were no significant three-way interactions of soil drought × wheat varieties × nitrate on leaf 1–5. In experiment 2 leaf length gradually increased with nitrate level, whereas in experiment 1 initially leaf length increased rapidly with nitrate level to an approximate asymptotic value between 2 and 5 mM nitrate (Fig. 3).

### 3.2 Tiller number and root growth

**Fig. 4:**
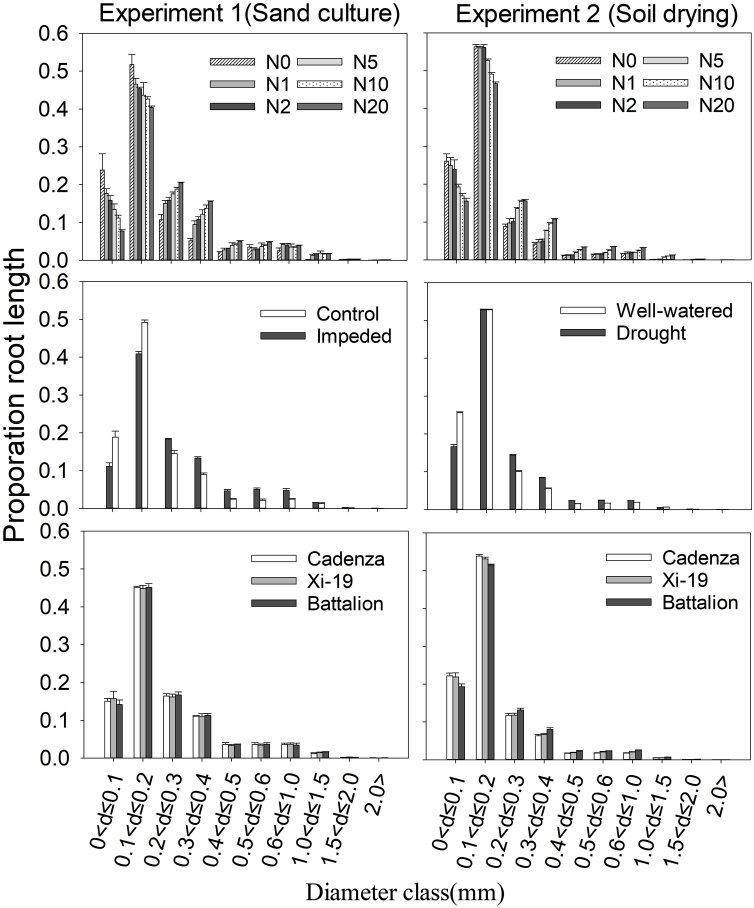
The distribution of root length with diameter for the three cultivars in the two experiments. The effect of nitrate concentrations on root diameter was significant at P < 0.001. Both the root impedance (sand culture) and drought treatments (soil drying) increased root diameter (P < 0.001).

The application of a root stress by either sand culture or soil drying always reduced the number of tillers, roots and the dry matter (Table 1, Table 2). The main effects of root impedance or drought and nitrate level on the tiller number, nodal root number and shoot and root dry weight were significant at *P* < 0.001 (Table 1, Table 2). There was significant three-way interaction of root impedance or drought × wheat variety × nitrate level on tiller number (*P* = 0.015, F_10,60_ = 2.45), shoot dry weight (*P* = 0.011, F_10,60_ = 2.61) and root dry weight (*P* = 0.028, F_10,60_ = 2.23) in the sand culture experiment, but only on nodal root number in soil drying experiment (*P* = 0.016, F_10,68_ = 2.36) (Table 2). The plants, which grew in the soil drying experiments, were much smaller than those, which grew in the sand culture. While the soil drying experiment was 30 days in duration compared with 40 days for the sand culture this does not account for the difference in the size of the wheat plants between the two experiments. Shoot dry matter in sand culture was approximately 10 times greater than those in the soil drying experiment in the control treatments, except for N0. These differences are, in part, due to the difference in tiller numbers and the number of nodal roots (Table 1, Table 2). In the drought experiment the roots were, apart from N0 and N1, approximately a third the length of those in the sand culture experiment. The roots that grew in the N0-drought treatment were longer than those in the N0-sand culture treatment (Table 3), which was probably due to residual nitrate in the loamy sand. Root diameter increased with impedance (*P* < 0.001, F_1,60_ = 195.65) and with drought (*P* < 0.001, F_1,60_ = 296.45) (Table 3 and Fig. 4). Root diameter always increased with nitrate level (*P* < 0.001, F_5,68_ = 64.00 in sand culture and *P* < 0.001, F_5,68_ = 171.25 in soil drying). At the higher nitrate levels (N10 and N20), total root length was lower (P < 0.001) (Table 3).

**Table 1:** The effect of root impedance and nitrogen supply in sand culture on the number of tillers, number of nodal roots, shoot and root dry weight at the point of harvest. The interaction among root impedance, nitrogen levels and cultivar is also shown. Each value is the mean of three replicates. ANOVA was conducted, *P*-values for root impedance, nitrogen levels, cultivar and their interaction are reported.

		Number of tillers		Number of nodal roots		Maximum root depth(cm)		Shoot Dry weight(g)		Root Dry Weight(g)	
Cultivar	N rates	control	impeded	control	impeded	control	impeded	control	impeded	control	impeded
	N0	4.7	0.7	16.0	9.7	39.7	20.0	0.4	0.08	0.3	0.1
	N1	11.0	6.0	45.3	26.3	27.6	19.8	3.9	2.6	1.4	0.8
	N2	20.3	9.0	60.3	41.0	33.6	18.7	9.4	5.4	2.4	1.8
Cadenza	N5	29.7	21.3	82.3	52.3	39.5	24.7	14.8	10.1	5.1	2.7
	N10	37.0	16.6	86.5	48.5	43.0	21.6	19.6	7.9	5.2	2.0
	N20	37.3	19.3	87.7	43	43.0	20.2	18.3	8.2	5.5	2.0
	N0	3.0	0.7	15.0	9.0	42.3	30.7	0.3	0.1	0.4	0.1
	N1	12.0	6.3	39.3	27.3	32.0	22.2	3.8	2.5	1.5	0.9
Xi-19	N2	17.7	10.3	51.7	34.7	36.3	19.3	6.8	4.8	2.2	1.3
	N5	29.7	21.0	92.3	49.3	43.7	25.8	14.2	8.7	4.6	2.2
	N10	33.0	24.0	86.6	54.3	42.5	33.0	15.8	10.6	5.0	2.4
	N20	34.3	21.0	85.5	45.0	38	24.7	16.4	9.4	4.2	2.0
	N0	2.0	0.7	12.7	10.3	39.0	34.2	0.2	0.1	0.2	0.2
	N1	15.6	8.7	39.7	29.3	31.8	24.7	3.6	2.1	1.4	0.8
	N2	23.0	14.7	55.3	37.1	36.5	26.5	6.8	4.7	2.5	1.3
Battalion	N5	32.7	27.7	78.2	54.3	38.0	31.8	13.2	10.1	4.6	2.4
	N10	31.0	24	77.0	48.5	36.7	26.3	13.4	10.7	4.0	2.2
	N20	32.7	25	70.7	47.0	33.3	26.0	11.8	8.8	3.1	2.0
P-value											
Nitrogen			<0.001		<0.001		<0.001	<0.001			<0.001
impedance			<0.001		<0.001		<0.001	<0.001			<0.001
cultivar			<0.001		0.137		0.040	<0.001			0.005
nitrogen×impedance			<0.001		0.613		0.621	<0.001			<0.001
nitrogen×cultivar			0.009		0.029		0.546	0.067			0.061
impedance×cultivar			0.009		0.581		0.004	<0.001			0.021
nitrogen×impedance×cultivar			0.015		0.317		0.692	0.011			0.028

**Table 2:** The effect of soil drought and nitrogen supply on the number of tillers, number of nodal roots, shoot and root dry weight at the point of harvest. The interaction among root impedance, nitrogen levels and cultivar was also shown. Each value is the mean of three replicates. ANOVA was conducted, P values for root impedance, nitrogen levels, cultivar and their interaction are reported.

		Number of tillers		Number of nodal roots		Shoot Dry weight(g/plant)		Root Dry Weight(g/plant)	
Cultivar	N rates	Well Watered	Drought	Well Watered	Drought	Well Watered	Drought	Well Watered	Drought
	N0	2.3	1.7	13.7	11.3	0.32	0.30	0.37	0.33
	N1	3	2.7	17.0	14.3	0.49	0.37	0.44	0.38
	N2	4.7	3	19.3	14.3	0.70	0.51	0.58	0.49
Cadenza	N5	8.0	5.3	26.3	17.7	1.20	0.75	0.84	0.68
	N10	14.0	8.0	32.0	19.3	1.79	0.99	0.95	0.55
	N20	13.0	8.0	32.0	19.0	1.75	0.99	0.53	0.41
	N0	2.7	2.3	13.0	10.3	0.35	0.29	0.44	0.42
	N1	3.3	3.0	15.3	12.3	0.49	0.39	0.52	0.43
Xi-19	N2	4.7	3.0	15.7	13.0	0.60	0.45	0.60	0.58
	N5	7.7	5.7	23.3	15.3	1.19	0.71	0.88	0.68
	N10	14.3	8.0	26.7	20.3	1.69	1.04	0.80	0.61
	N20	15.7	9.3	27.0	22.0	1.69	1.18	0.48	0.46
	N0	2.3	2.3	12.7	11.0	0.32	0.30	0.37	0.39
	N1	4.0	3.0	14.0	12.3	0.48	0.38	0.48	0.41
	N2	4.7	3.7	16.0	13.0	0.68	0.53	0.64	0.50
Battalion	N5	7.3	6.0	22.3	15.3	1.12	0.72	0.77	0.63
	N10	13.7	8.3	26.3	20.3	1.45	1.00	0.65	0.63
	N20	14.0	8.3	27.0	20.3	1.61	1.02	0.50	0.46
*P*-value									
nitrogen			<0.001		<0.001	<0.001			<0.001
water treatment			<0.001		<0.001	<0.001			<0.001
cultivar			0.014		<0.001	0.083			0.019
nitrogen× water treatment			<0.001		<0.001	<0.001			<0.018
nitrogen×cultivar			0.125		0.702	0.516			0.213
Water treatment ×cultivar			0.510		<0.001	0.181			0.390
nitrogen× water treatment ×cultivar			0.848		0.016	0.695			0.065

**Table 3:** The effect of root impedance, drought treatment and nitrogen on root diameter and length. Each value is the mean of three replicates. The SED is 0.023 and 60.5 for root diameter and root length in the sand culture experiment. The SED (standard error of differences) is 0.01 and 13.2 for root diameter and root length in the pot experiment, respectively.

		Root diameter(mm)				Total Root length(m)			
Cultivar	N rates	control	impeded	Well Watered	Drought	control	impeded	Well Watered	Drought
	N0	0.201	0.239	0.174	0.202	56.6	17.9	79.8	77.2
	N1	0.235	0.295	0.175	0.204	148.5	78.7	105.8	83.2
	N2	0.241	0.326	0.184	0.209	301.2	120.5	133.2	97.7
Cadenza	N5	0.255	0.333	0.211	0.251	558.9	165	161.3	111.4
	N10	0.264	0.344	0.230	0.270	595.4	141.5	181.0	99.3
	N20	0.299	0.350	0.254	0.309	545.7	122.1	98.4	59.3
	N0	0.205	0.233	0.185	0.205	51.8	17.6	92.4	86.8
	N1	0.236	0.303	0.192	0.211	184.7	85.1	112.5	87.0
Xi-19	N2	0.243	0.337	0.192	0.224	263.0	110.7	131.8	103.1
	N5	0.255	0.344	0.231	0.246	539.3	181.3	156.6	119.7
	N10	0.264	0.345	0.236	0.272	640.6	266.7	161.6	103.8
	N20	0.299	0.359	0.246	0.295	484.5	161.6	95.9	77.1
	N0	0.209	0.250	0.199	0.220	38.6	19.8	76.8	76.0
	N1	0.236	0.309	0.207	0.230	170.1	68.3	98.5	71.0
	N2	0.242	0.313	0.204	0.231	271.8	128.2	126.8	85.8
Battalion	N5	0.266	0.321	0.227	0.263	499.8	184.3	140.1	95.6
	N10	0.274	0.345	0.256	0.298	468.3	174.9	126.7	93.5
	N20	0.300	0.362	0.260	0.320	319.9	142.1	89.7	65.3
*P*-value									
Nitrogen			<0.001		<0.001		<0.001	<0.001	
Impedance/drought			<0.001		<0.001		<0.001	<0.001	
Cultivar			0.726		<0.001		0.037	<0.001	
nitrogen × impedance/drought			0.050		<0.001		<0.001	<0.001	
nitrogen × cultivar			0.998		0.215		0.274	0.736	
Impedance/drought × cultivar			0.814		0.240		0.048	0.288	
nitrogen × impedance/drought × cultivar			0.989		0.928		0.649	0.737

### 3.3 Soil drying and water uptake in experiment 2

**Fig. 5:**
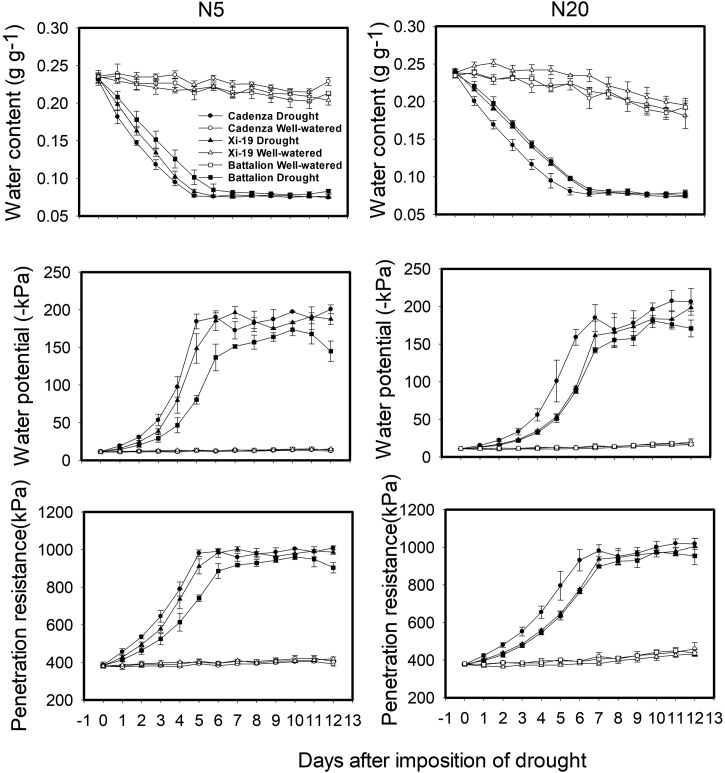
Water content, matric potential and penetrometer resistance in the 5 and 20 mM nitrate treatments of the sol drying experiment. The water content was determined by weight and the matric potential and penetrometer resistance were determined from a water release curve and from a calibration of penetrometer resistance against soil water content.

Soil water content as a function of time for the N2 and N20 treatments is shown in Fig. 5 along with matric potential data determined from the water release characteristic and penetrometer resistance determined from a calibration against water content (the complete data set is given in Fig. S1). Following the imposition of the drought treatment the rate of soil drying increases with the nitrate concentration, due increased plant growth. In the drought treatment, the matric potential at the lower water content depended on the nitrate concentration. At N0 the final matric potential was approximately −90 kPa compared with −175 kPa for the N20 treatment. However, these differences corresponded to very small differences in soil water content. The final penetrometer resistance was approximately 1 MPa and also depended on the nitrate concentration; at N0 penetrometer resistance was approximately 0.9 MPa compared with 1 MPa at N20. There were differences in the rate of soil drying by the different wheat varieties; Cadenza seemed to dry the soil at the greatest rate (Fig. S1). This was most clearly seen from the matric potential and penetrometer data. The cumulative transpiration data following the application of the drought treatment are shown in Fig. S2. In the well-watered treatment, transpiration was very sensitive to the nitrate level (*P* < 0.001, F_5,68_ = 140.75). In the drought treatment transpiration was relatively insensitive to nitrate treatment.

### 3.4 Nitrogen uptake

**Fig. 6:**
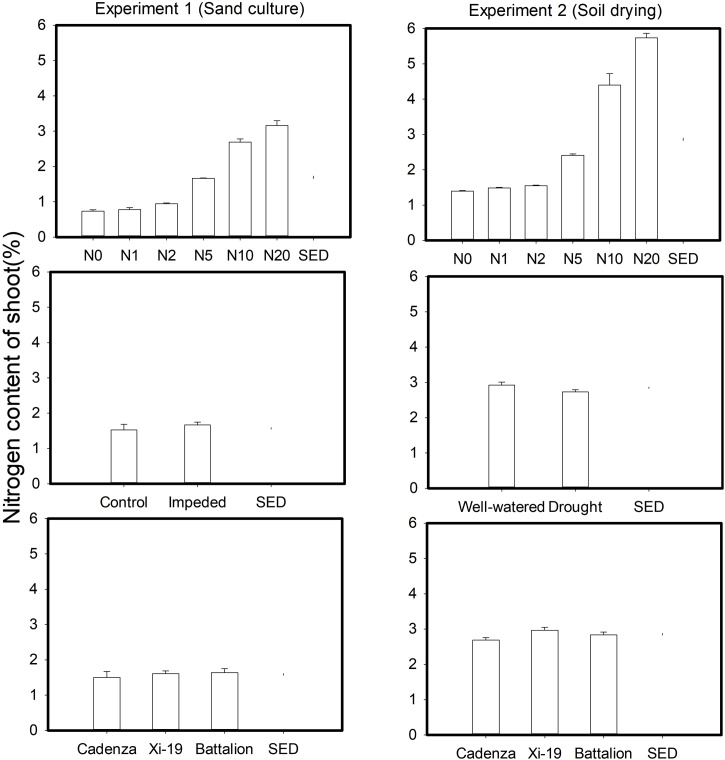
The main effects of nitrogen supply, root impedance/drought and cultivar on the percentage nitrogen concentration of shoot. There was no interaction between these treatments and the effect of the treatments on nitrogen concentration of shoots is fully described by the main effects (P < 0.001).

The nitrogen concentrations of the leaves at harvest are shown in Fig. 6. No significant three-way interactions of root impedance/drought × wheat varieties × nitrate were observed for leaf N concentration in either experiment. The concentration of leaf N increased with the nitrate concentration (*P* < 0.001, F_5,68_ = 162.57 in sand culture and *P* < 0.001, F_5,68_ = 497.67 in soil drying). The N concentration in plants grown in sand culture was smaller than that of those in the drought experiment. The higher N concentration in the plants grow in the drought experiment may be related to the requirement for frequent irrigation, which used nutrient solutions, to maintain soil water status. However, the total N shoot uptake of the soil grown plants was approximate 25% of the plants grown in sand culture. In the sand culture experiment, root impedance increased the N concentration of the leaves from 1.52 to 1.66% (*P* = 0.027, F_1,60_ = 5.15). However, the drought treatment decreased the leaf N concentration relative to the well-watered plants from 2.93 to 2.73% (*P* < 0.001, F_1,60_ = 33.64). A significant effect of wheat varieties on leaf N was only found in the soil drying experiment (*P* < 0.001, F_2,60_ = 21.00)

**Fig. 7:**
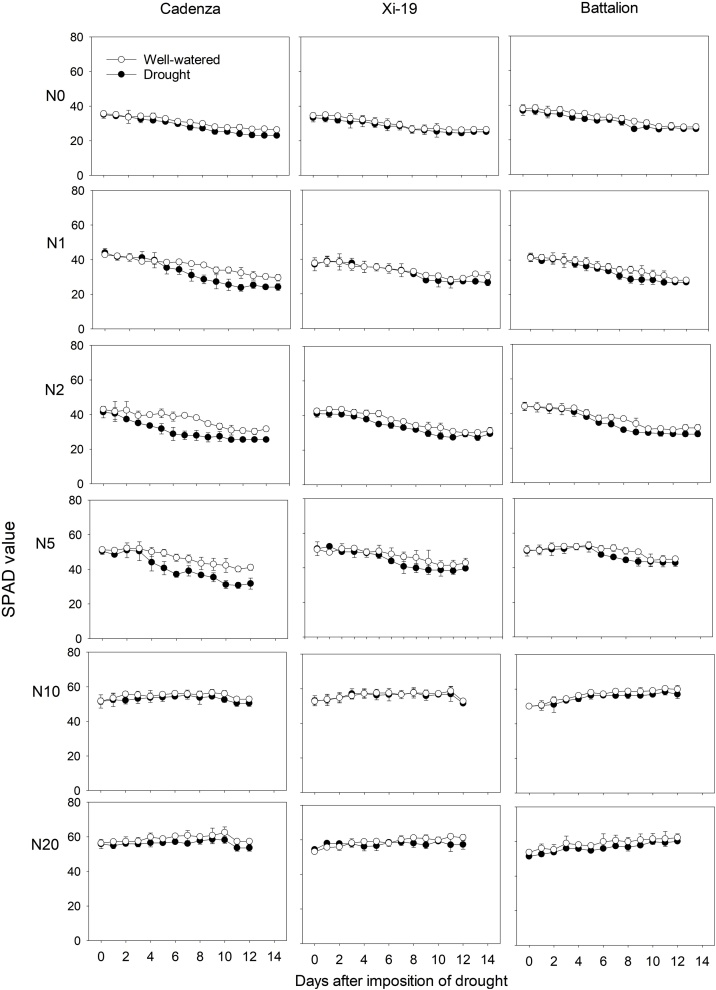
The SPAD value of leaf 3 in three cultivars under well-watered and drought treatment in the soil pot experiment. The plots show the mean SPAD value. The standard errors of the means are shown.

**Fig. 8:**
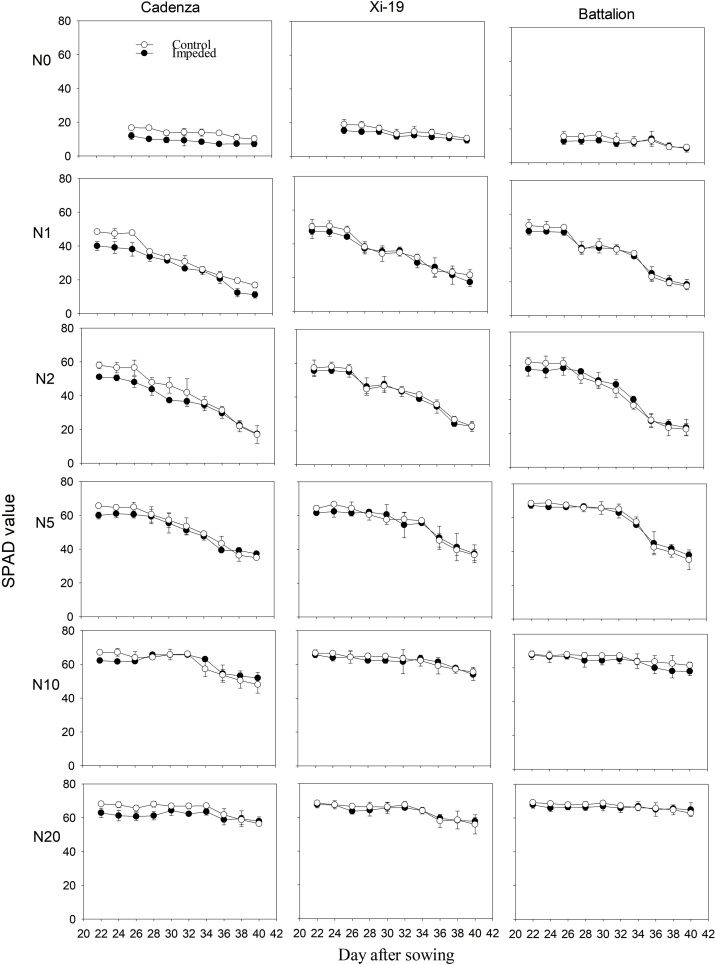
The SPAD value of leaf 3 in three cultivars under control and root impeded treatment in the sand culture experiment. The plots show the mean SPAD value. The standard errors of the means are shown.

Leaf SPAD data (leaf 3) are correlated with the N concentration in the shoots (Lopez-Bellido et al., 2004) and are shown in Fig. 7, Fig. 8. SPAD values in the drought experiment (Fig. 7) decreased with time in the Low N treatments (N0 to N5) but remained constant in the higher N treatments (N10 and N20). SPAD values showed little treatment effects at N10 and N20. The SPAD data from the sand culture experiment (Fig. 8) were similar to those data from the drought experiment.

## 4 Discussion

### 4.1 Plant architecture

Our data from the sand culture experiment confirm our previous finding (Jin et al., 2015a) that leaf elongation in Cadenza, which has a tall *Rht* allele appears to be more sensitive to the effects of root impedance than semi-dwarf wheats. In the nitrate treatments, N > 10 mM, the final leaf length of Cadenza with root impedance was 82% of the control, whereas the length of leaves of Xi19 and Battalion, when impeded, were approximately 92% of the control. Leaf elongation seemed to be insensitive to the nitrate treatment between 5 and 20 mM nitrate (N5 to N20) (Fig. 3). In Cadenza the leaf length was greatly decreased by root impedance at all nitrate treatments, but the leaf stunting in Xi19 and Battalion by root impedance was less sever (Fig. 3).

Leaf stunting was less severe in the drought experiment (Fig. 3). However, in this treatment all plants were initially well-watered until the emergence of leaf 5, when in the drought treatment, watering was limited; this occurred approximately at day 14. In the “drought” treatments the soil dried to an equilibrium water content (7.5 g cm^−3^), the estimated matric potential was approximately −200 kPa, and the penetrometer resistance was approximately 1 MPa (Figs. 5 and S1). The penetrometer resistance is comparable with the penetrometer resistance in the impedance treatment of the sand culture experiment (Whalley et al., 1999).

Both root impedance and drought treatment resulted in fewer nodal roots (*P* < 0.001 in both experiments), a smaller total root length (*P* = 0.037 in sand culture and *P* < 0.001 in soil drying) and lower root dry weight (Table 1, Table 2). Root diameter increased (*P* < 0.001) in both experiments (Table 1, Table 2, Table 3). Increased root diameter is commonly reported response to root impedance (Clark et al., 2008; Whiteley and Dexter, 1981). When maize roots are exposed to a water stress of -1.6 MPa, in vermiculite, in the absence of root impedance, they become thinner (Liang et al., 1997), while the diameter of pea roots grown in polyethylene glycol is insensitive to water potential between 0 and -1.0 MPa (Whalley et al., 1998). Short term water stress greater than approximately -1.00 MPa decreases the diameter of wheat roots (Whiteley and Dexter, 1981). Root diameter increased with both root impedance in the sand culture as well as in the drought treatment (Fig. 4; Table 3); thus, it is likely to be a response to root impedance in both experimental systems. We also found that root diameter increased with nitrate concentration in both experimental systems. Darwent et al. (2003) also found that increased shoot nitrate concentration was correlated with an increased mean root diameter and considered that this response was consistent with increased production of fine roots under low N-supply.

Impedance resulted in shorter roots (Table 1) which is consistent with previous reports using the same sand culture system (Coelho Filho et al., 2013; Jin et al., 2015a; Whalley et al., 2006). The position of the capillary fringe in our experiments (Fig. 1) may have restricted rooting depth, which can be much greater when roots are grown in unsaturated soil in deep rhizotrons (Jin et al., 2015a,b; Manschadi et al., 2006, 2008). Wheat root growth is greatly affected by the presence of a water table, although the unimpeded roots elongated below the capillary fringe (see Fig. 1) and into the saturated sand, for example the rooting depth of Cadenza was up to 43 cm in the control, which extends into saturated sand. It is probably inadvisable to draw general inferences about rooting depth data obtained in sand culture systems. In comparable sand culture experiments measurements of oxygen diffusion suggest that this is not limiting (Whalley et al., 1999). In the both the sand culture and soil drying experiments total root length was lower at both low and high nitrate levels (Table 3).

In Arabidopsis, the number of lateral roots increased with both water and nitrate availability (Chapman et al., 2011). In this work, we found that the number of nodal axes increased with nitrate availability (Table 1, Table 2). We also found the number nodal axes was lower in the soil grown treatment with limited watering. Increased root impedance also has the effect of lowering the number of nodal axes. There is a close correlation between tiller number and the number of nodal axis that holds irrespective of the whether the stress is nutrient or abiotic (Fig. S3). We found no other coordination between the other plant architectural parameters. In rice the nitrate transporter OsNPF7.2 has been implicated in the coordination of the cytokinin and strigolactone pathway and the regulation of tiller number (Wang et al., 2018). Although root impedance affects tiller number and the number of root axis in both wheat (Coelho Filho et al., 2013) and rice (Clark et al., 2002), wheat differs from rice in that yield is not closely related to the number of tillers on each plant. In wheat, yield is related the number of grain bearing heads, which is determined by both seed rate and tiller number. Nevertheless, it appears in wheat that nitrate availability and abiotic stress are likely to jointly coordinate an, as yet, unidentified hormone signalling pathway that is responsible for the number of tiller per plant.

In common with many studies (e.g. Roycewicz and Malamy, 2012), we found that at higher nitrate levels (N10 and N20), there was inhibition to root elongation. However, we did find that there was an interaction between the effects of root impedance/drought and nitrate level (*P* < 0.001) on root length. When roots were impeded or droughted the inhibitory effects of high nitrate levels on root elongation were less clear.

### 4.2 Water uptake in experiment 2

The water stress applied in the soil drying experiment was not that great compared to studies, which use vermiculite or polyethylene glycol (Verslues et al., 1998), where the water potential can be as low as -1.6 MPa. However, the hydraulic conductivity of the soil was reduced by several orders of magnitude at the lower water content (Fig. S4) although the mean diffusivity of this soil is approximately 0.61 cm^2^ day^−1^ (Choudhury et al., 2018). The final root length densities were high and ranged from 17 to 30 cm cm^−3^, which is comparable with those found in the surface layers of soil in the field (White et al., 2015; Hodgkinson et al., 2017), and are sufficiency high to completely dry the available water in soil (Deery et al., 2013; Gregory et al., 1978a, 1978b). When both root length density and the hydraulic conductivity of soil are high there should be no limitation to water uptake; this was the case in our experiment and soil drying should be simply related to shoot size and root length density (i.e. the size of the plant sink strength). We found that the final transpiration rate, T (g), was empirically related to shoot biomass, S, and root length density, R by

T=-13.41(±3.04)+20.82 (± 1.96)S+0.108(±0.032)R

Where S is in grams and R is in cm cm^−3^. This explained 85 percent of the variance in T (P < 0.001). Including soil moisture treatment in a grouped regression, to take account of differences in soil conductivity, increased the percentage variance accounted for to 96 percent. Although this was a statistically significant increase (i.e. from 85% to 96.0% P < 0.001), it is clear that it is the plant and not the soil that predominately determines soil drying, with differences in soil conductance between the two treatments being of secondary importance. Simple regression against either S or R explained 80.8 and 36.9 percent of the variance in T respectively (both at P < 0.001). When using grouped regression to take account of soil moisture S explained 96 percent of the variance while R explained 45 percent of the variance (P < 0.001 for both S and R). The comparatively low percentage of variance accounted for by root length density alone is almost certainly because in all treatments root length density was high and the soil was conductive.

Root permeability depends on nitrate levels (Gorska et al., 2008). Li et al. (2016) report a strong correlation between nitrate accumulation in the shoots and root hydraulic conductivity. The high nitrate status of the soil grown plants compared with those grown in the sand culture (Fig. 6) may be associated with a higher root conductance and this might party explain the comparatively low percentage of variance accounted for by root length density alone. However, in this study it is not possible to separate out the effects of nitrate on any possible increase in root conductance as opposed to increased shoot growth (i.e. increased sink strength). Nevertheless, Deery et al. (2013) identified changes to plant hydraulic resistance as factor that could explain differences in water uptake in experiments similar to experiment 2.

Our data highlight a key point in the crop growth in the field; in densely rooted surface layers soil drying to moderate water potentials (e.g. −200 kPa) is largely unaffected by soil hydraulic properties. Previously, we have shown that yield losses in the field in drying soil are more likely to be due to the associated increase in soil strength rather than water availability (Whalley et al., 2006; Whitmore et al., 2011). That our soil drying experiment only modified water availability indirectly through the effect on shoot and root growth adds weight to the viewpoint that the primary effect of moderate soil drying is due to increased soil strength, restricting root elongation and stunting leaf elongation. Furthermore, it adds weight to our earlier speculation the root thickening and leaf stunting we have observed are related to effects of root impedance and not water availability.

### 4.3 Nitrogen uptake

In both experiments at nitrate concentrations of 10 and 20 mM, SPAD values did not decrease greatly with time (Fig. 7, Fig. 8). At lower nitrate concentrations in the sand culture system, SPAD values deceased with time quicker than in the irrigated soil grown plants. This is likely to be due to the higher nitrate concentration in the shoots of the soil grown plants (Fig. 6). The nitrate nitrogen concentrations in the shoots (Fig. 6) are comparable with those found in post-anthesis plants in the field, which can range from 1.5 to 4.5% and correspond to SPAD reading from 30 to 55 (Lopez-Bellido et al., 2004). Even though the nitrogen content of the soil was low, comparison of the N0 treatments in the experiments 1 and 2, showed that in soil the limited nitrogen was accessible to the roots. The nitrogen concentration of the shoots grown in N0 in soil was approximately double that of those grown in sand and they showed greater leaf elongation (Fig. 3). A key finding of this work is that the leaf length is stunted irrespective of nitrate supply or nitrogen status of the leaf. A practice implication is the most effective use of nitrogen applied to crops can only be obtained in soils which do not strengthen greatly as they dry; these are soils with high clay or organic matter content (Whalley et al., 2007). Our data suggest the increased nitrogen fertilization will not work as a strategy to offset the stunting effects of root impedance.

## 5 Conclusions

We have compared the growth of wheat exposed to root impedance either by a confining pressure in a sand culture experiment or by soil drying, at a range of nitrate availabilities. Leaf stunting occurred irrespective of nitrate supply. Leaf elongation in Cadenza was more sensitive to root impedance than semi-dwarf wheats (Xi19 and Battalion), which confirmed our previous findings. In both experimental systems root diameter increased which is a widely-reported response to increased root impedance. Root diameter increased with nitrate concentration. This appears to be due to a greater number of fine roots at low nitrate levels. We found that the number of tillers was highly correlated with the number of nodal roots with little evidence of any genotypic effects. The number of nodal roots decreased with decreasing nitrogen and water availability and increased root impedance. In our soil drying experiment, our data suggest that water uptake was not limited by the soil hydraulic properties, but closely related to shoot size and rooting density. This implies that in our pot experiment plant, with drying soil, growth was limited by root impedance.

## Conﬂicts of interest

The authors declare no conﬂicts of interests.
