# Supplementary material for: Multiple abiotic stress, nitrate availability and the growth of wheat
Source: Soil Tillage Res. 2019 Aug;191:171–84. doi: 10.1016/j.still.2019.04.005 (PMC6559134; doi:10.1016/j.still.2019.04.005)
Supplement: Supplementary file 1 [file mmc1.docx]

Supplemental data

The effects of root impedance, water availability and nitrogen availability on the early growth of wheat: a comparison of two growth systems

Y. Ge, M.J. Hawkesford, C.A. Rosolem, S.J. Mooney, R.W. Ashton, W.R. Whalley

Fig. S1. Development of soil water content, soil water potential and penetration resistance of well-watered and drought treatments. The plots show the mean soil water content, water potential and penetration resistance. The standard errors of the mean are shown.

Fig.S2. Cumulative transpiration per plant in three wheat cultivars under two water treatments and six nitrogen supply levels. The main effects (water treatment, cultivars and nitrate concentrations) had a significantly effect at *P*<0.001.

Fig. S3. The relationship between the number of tillers and nodal roots across all the experiments.

Fig. S4. The hydraulic conductivity of Butt Close soil as a function of matric potential estimated with the Hyprop instrument (METER Group, Inc. USA)

Fig. S1

Fig. S2

Fig. S3

Fig S4
